# Effects of dietary genistin supplementation on reproductive performance, immunity and antioxidative capacity in gestating sows

**DOI:** 10.3389/fvets.2024.1489227

**Published:** 2024-11-21

**Authors:** Yuchen Li, Bing Yu, Chunxue Liu, Shuangshuang Xia, Yuheng Luo, Ping Zheng, Guanglei Cong, Jie Yu, Junqiu Luo, Hui Yan, Jun He

**Affiliations:** ^1^Institute of Animal Nutrition Sichuan Agricultural University, Chengdu, China; ^2^Anyou Biotechnology Group Co., Ltd., Taicang, China

**Keywords:** genistin, gestation sow, reproductive performance, serum biochemistry, placental gene

## Abstract

Genistin is an isoflavone of soybean, with estrogenic activity. This experiment was conducted to investigate its effect on reproductive performance, antioxidant capacity, and immunity in gestating sows. Seventy-two sows (Landrace × Yorkshire) were selected and randomly divided into two treatment groups (*n* = 36) based on their backfat thickness, parity and fed with basal diet or supplementation of 150 mg/ kg genistin to the basal diet based on DMI for the entire gestation period. Results showed that dietary genistin supplementation significantly increased the average number of live born per litter (*p* < 0.05), and tended to increase the number of healthy piglets per litter (*p* = 0.058), but decreased the average weight of live born per litter (*p* < 0.05). Dietary genistin supplementation significantly decreased the number of mummified and stillbirths per litter (*p* < 0.05). Moreover, the average daily feed intake (ADFI) and total feed intake of the gestating sows were also increased in the genistin-supplemented group (*p* < 0.05). Genistin significantly increased the serum concentrations of catalase (CAT), immunoglobulin A (IgA), IgG, and IgM at 35 days of gestation (*p* < 0.05). The serum concentrations of interleukin-10 (IL-10) and interferon-γ (IFN-γ) were also increased upon genistin supplementation (*p* < 0.05). However, genistin supplementation tended to decrease the serum concentrations of total cholesterol (TC), low-density lipoprotein cholesterol (LDL-C), and leptin at 85 days of gestation (*p* = 0.081 and *p* = 0.096, respectively). Interestingly, genistin supplementation decreased the transcript abundance of interferon-γ (IFN-γ) and placental imprinting gene H19, but significantly increased the transcript abundance of insulin-like growth factor I (IGF-I) and amino acid transporters such as the sodium-coupled neutral amino acid transporter 2 (SNTA2) and SNAT4 in the placenta (*p* < 0.05). These results suggested that dietary genistin supplementation during gestation can improve the reproductive performance of sows, which was probably associated with improving of antioxidant capacity and immunity, as well as changes of transcript abundance of critical functional genes in the placenta.

## Introduction

The number of stillbirths, mummies, abortions, and early embryonic death has a significant impact on both the reproductive performance metrics of sows and the economic viability of farms (1). Previous studies indicated that the mortality rate of pre-implantation embryos in sows falls within the range of 20 to 50%, with the majority of losses occurring between 12 to 18 days post-breeding, during the embryonic implantation period (2–4). Furthermore, a separate study underscores that over 70% of embryonic fatalities occur within the initial 25 days of gestation (5).

In the intricate choreography of mammalian reproduction, a diverse array of hormones played a pivotal role. Among these, estrogen, which was secreted by the ovary and placenta, held particular significance. It was instrumental in inducing estrus, fostering oocyte maturation, sustaining gestation, and triggering parturition mechanisms in the later stages of gestation (6–8). Prior research had shown that sows in their second to fifth parity demonstrated superior reproductive performance (9). However, as the number of mammalian litters increases, it was often accompanied by a lack of estrogen production, leading to a rapid decline in mammalian reproductive performance (10). Studies had demonstrated that estrogen supplementation could effectively enhance the physiological state and reproductive capabilities of animals. For example, studies have found that estrogen could improve the physiological state of menopausal mice, increase body weight and feed intake, and increase vitality (11). Another study showed that estrogen could promote the development and activation of chick primordial follicles (12).

Phytoestrogens have similar chemical structure as natural estrogens and can bind to estrogen receptors to exert estrogenic function (13). Phytoestrogens may be divided into chalcones, flavonoids (flavones, flavonols, flavanones, isoflavones), lignans, stilbenoids and miscellaneous classes according to their chemical structure and biosynthesis (14, 15). The isoflavonoids has attracted more attention than other groups, as they are abundant in a wide variety of natural plants (16). Recent studies have shown that animal reproductive performance can be improved by dietary supplementation of various phytoestrogens. For instance, daidzein and genistein are two major components of isoflavones. Daidzein could improve the reproductive performance of female Zhedong White geese by altering serum hormone concentrations (17). Genistein has been reported to improve the reproductive performance of breeder hens during the late egg-laying period (18) and might exhibit therapeutic potential for polycystic ovarian syndrome mice the ER-Nrf2-Foxo1-ROS pathway (19). However, an extremely high dose of genistein may lead to infertility in female mice (20, 21). Although, numerous studies investigated the influence of phytoestrogens on animal production, there are few reports on the application of genistin in sows. Therefore, this study was aimed to investigate the effects of dietary supplementation with genistin during gestation on reproductive performance, antioxidant capacity, and immunity of sows.

## Materials and methods

### Animals and experimental design

A total of 72 multiparous sows (3 to 6 parity, Yorkshire × Landrace) with an average backfat thickness of 13.79 ± 2.45 mm were selected from Sanmenxia Tianyuan swine farms. From the first day of artificial insemination, the sows were randomly divided into two groups (*n* = 36); (I) control group [basal diet (BD)], (II) genistin group (BD was supplemented with 150 mg/kg genistin). Genistin was provided by Guilin Fengpeng Biotechnology Co., Ltd. (Guilin, China), purity: 10% soybean isoflavones, genistin accounted for 8.5%. Before the start of the trial, genistin was thoroughly blended with the premix and subsequently incorporated into the compound feed to ensure even distribution, resulting in a genistin concentration of 150 mg/kg in the feed. One week prior to parturition, the sows were transferred from the gestation house to the farrowing room. Five sows in the control group and two sows in the genistin group were empty or had an abortion.

### Diets and feeding management

The nutritional requirements recommended by the National Research Council (U.S.) in 2012 were met or slightly exceeded by the basic diet (Table 1). In this experiment, the concentration of genistin in the basal diet was 24.7 mg/kg (detected by Qingdao Kechuang Quality Testing Co., Ltd.). Gestation sows are raised in a single column in the gestation house, and the gestation house adopts a full fecal floor. The sows were fed twice a day (7:30 and 15:00), 1.8–2.0 kg/d (1–30 d); 2.3–2.7 kg/d (31–85 d); 3–3.5 kg/d (86–114 d). The gestation house was kept ventilated and breathable, dry and clean, and the room temperature was controlled at 20–25°C. Sows had *ad libitum* access to drinking water. Management and treatment plans were performed according to the swine farm plan.

**Table 1:** The composition and nutrient level of the diets used for the experiment.

Ingredients	%	Nutritive value	Calculated nutrient concentrations, %
Corn	37	Crude protein	13.8
Wheat middling	15	Crude fat	6.3
Dehulled soybean meal 46	7.5	Crude fiber	5.5
Wheat bran	15	Available calcium	0.8
Full-fat rice bran	10	Available phosphorous	0.33
Extruded flaxseed	1	Salt	0.45
Soybean hull	9.5	SID-Lys	0.68
Soybean oil	1	NE, MJ/kg	9.62
Premix^1^	4		
Total	100		

1 The premix provides the following per kilogram of diet: vitamin A, 8,000 IU; vitamin D, 32,000 IU; vitamin E, 40 mg; vitamin K, 2 mg; vitamin B1, 3 mg; vitamin B2, 5 mg; vitamin B6, 3 mg; vitamin B12, 0.04 mg; niacin, 30 mg; pantothenic acid, 20 mg; folic acid, 1.5 mg; biotin, 0.3 mg; riboflavin, 8 mg; Fe, 100 mg; Cu 20 mg; Zn, 80 mg; Mn, 40 mg; I, 0.3 mg; Se, 0.25 mg.

### Sample collection and farrowing record

At 7:00 in the morning of 35 and 85 days of gestation, 6 sows with similar average backfat thickness and parity were randomly selected from each group. These sows were fasted, and the standing Baoding method was employed to secure them with Baoding ropes, ensuring both their safety and comfort. Subsequently, blood samples were obtained via venipuncture of the anterior vena cava. The collected blood was allowed to stand at room temperature for 30 min. Thereafter, serum separation was accomplished by centrifugation at 3,500 r/min for 15 min and stored at −20°C for later analysis.

To calculate the average weight of piglets per litter and average weight of live born piglets, the weight of each piglet was recorded during farrowing (before drinking colostrum). The number of piglets per litter (including total born, live born, healthy piglets, weak piglets, and mummified and stillborn) was recorded. Healthy piglets refer to piglets with birth weight ≥0.9 kg, and weak piglets refer to piglets with birth weight <0.9 kg (farm regulations). Sow natural childbirth. The farrowing duration was recorded. The duration of farrowing means the time from the birth of the first piglet to the expulsion of all placentas. The daily feed intake of each sow was recorded to calculate the total feed intake and average daily feed intake.

After delivery, 6 sows with successful delivery were selected from each group, and the placenta was washed with saline water. Placental tissues of 3 cm × 3 cm were collected in sterilized cryopreservation tubes, which was quickly frozen in liquid nitrogen and transferred to −80°C for storage.

### Analysis of serum hormone and metabolite contents

The contents of estradiol (E2, Item No. MM-0480O1), leptin (LEP, Item No. MM-0395O1), progesterone (PROG, Item No. MM-1205O1) and insulin growth factor-1 (IGF-1, Item No. MM-78075O1) were analyzed using the respective enzyme-linked immunosorbent assay (ELISA) kits (Jiangsu Meimian Industrial Co., Ltd., China) following the manufacturer’s instructions; and the serum concentrations of total protein (TP), albumin (ALB), glucose (GLU), blood urea nitrogen (BUN), triglyceride (TG), total cholesterol (TC), high-density lipoprotein cholesterol (HDL-C) and low-density lipoprotein cholesterol (LDL-C) were determined using Hitachi Automatic Biochemical Analyzer 3100 (Hitachi Diagnostic Products Co., Ltd., Shanghai). The minimal detection limits for E2, PROG, LEP and IGF-1 were 8 pmol/L, 80 pmol/L, 80 ng/L and 3 ng/L, respectively. And the intra-assay coefficient of variation (CV) of all kits was 10%, and the inter-assay CV was 12%.

### Analysis of serum immunoglobulins and cytokines contents

The contents of immunoglobulin A (IgA, Item No. MM-0905O1), IgG (Item No. MM-0403O1), IgM (Item No. MM-0402O1), interleukin-1 (IL-1, Item No. MM-0426O1), IL-6 (Item No. MM-0418O1), IL-10 (Item No. MM-0425O1), interferon-γ (IFN-γ, Item No. MM-0412O1) and tumor necrosis factor-α (TNF-α, Item No. MM-0383O1) were analyzed using the respective enzyme-linked immunosorbent assay (ELISA) kits (Jiangsu Meimian Industrial Co., Ltd., China) following the manufacturer’s instructions. The lowest detectable levels of kit IgA, IgG, IgM, IL-1, IL-6, IL-10, IFN-γ and TNF-α were 1 μg/mL, 1.2 μg/mL, 12 μg/mL, 3 ng/L, 50 ng/L, 8 ng/L, 100 pg/mL and 10 pg/mL, respectively, and the intra-assay coefficient of variation (CV) of all kits was 10%, and the inter-assay CV was 12%.

### Analysis of serum oxidant and antioxidant contents

The contents of superoxide dismutase (SOD, Item No. A001-2-2), total antioxidant capacity (T-AOC, Item No. A015-1-2), glutathione peroxidase (GSH-Px, Item No. A005-1-2), catalase (CAT, Item No. A0071-1) and malondialdehyde (MDA, Item No. A005-1-2) were analyzed using the respective assay kits (Nanjing Jiancheng Bioengineering Institute Co., Ltd.). All measurements were carried out in accordance with the manufacturer’s procedures.

### RNA extraction and quantitative real-time polymerase chain reaction (qPCR)

Total RNA was extracted from frozen placenta samples in strict accordance with the reagent instructions of RNAiso Plus (TaKaRa, Japan). The concentration and purity of total RNA were determined by a Thermo NanoDrop-ND2000 spectrophotometer. Samples with OD260:OD280 ratios ranging from 1.8 to 2.0 were regarded as suitable for cDNA synthesis. Additionally, we determined the RNA integrity of term placentas using 1% agarose gel electrophoresis, which showed 5S, 18S, and 28S rRNA bands. Moreover, the 28S:18S ribosomal RNA band ratio was found to be ≥1.8.

The RNA samples were reverse transcribed into cDNA using a reverse transcriptase HiScript III RT SuperMix for qPCR (+gDNA wiper) kit (Vazyme, Nanjing, China) with approximately 1.0 μL RNA sample by following the manufacturer’s protocols. Detection the glutamate cysteine ligase modified subunit gene *GCLM* in placental tissue; fatty acid transporter 1 (*FATP1*); glucose transporters gene *SLC2A3* and *SLC2A13*; amino acid transporter gene *SANT1*, *SNAT2 SNAT4* and *SLC38A10*; *insulin-like growth factors-1 (IGF1)* (22); placental imprinting genes *IGF2* and *H19*; placental pro-inflammatory cytokine gene *IL1-β* and *TNF-α*; the transcript abundance of antioxidant gene *SOD2* and *GSH-Px*. TB Green^®^ Premix Ex TaqTM II (Takara, Japan) reagent instructions are referred to for the operation steps and reaction system. The reaction system was 10 μL, containing TB Green premix Ex Taq II 5 μL, forward primer 0.4 μL, reverse primer 0.4 μL, ROX reference 0.2 μL, DEPC water 2 μL and cDNA 2 μL, The concentration of primers used was 10 μM. The thermal cycling conditions were as follows: initial denaturation at 95°C for 5 s, annealing at 60°C for 34 s and finally, extension at 72°C for 5 min. After amplification, a melting curve analysis was performed following each qPCR assay to check the specificity and purity of the resulting PCR products. The primers were consulted in the NCBI database and the primers of target gene sequence were synthesized by Sangon Biotech (Shanghai) Co., Ltd. The Primer and probe sequences are shown in the Table 2. The mRNA transcriptional abundance of the 2 groups were calculated using the $2^{-\Delta \Delta C_{T}}$ method with b-actin as the reference gene (23).

**Table 2:** Primers used for quantitative real-time PCR.

Genes	Primer and probe sequences (5′ to 3′)	Length, bp	Accession number
*IL1-β*	F: GAAAGCCCAATTCAGGGACC	90	NM_214055.1
	R: GGCGGGTTCAGGTACTATGG		
*TNF-α*	F: TTGAGCATCAACCCTCTGGC	123	NM_214022.1
	R: GGCATACCCACTCTGCCATT		
*IGF-1*	F: CTCTTCAGTTCGTGTGCGGAGAC	136	NM_214256.1
	R: TCCAGCCTCCTCAGATCACAGC		
*IGF-2*	F: CAGCCGTGGCATCGTGGAAG	170	NM_213883.2
	R: AGGTGTCATAGCGGAAGAACTTGC		
*H19*	F: TTCCTTGGAGGCTGTTCTGCT	109	XM_005659862.3
	R: ACGGTTTCTCATTTTGCCTTTAC		
*SNAT1*	F: GCAGGTCTTCGGCACCACAG	80	XM_003355629.4
	R: GGTAGCTCAGCATTGCTCCAGTG		
*SNAT2*	F: GCCGCAGCCGTAGAAGAATGATG	125	NM_001317081.1
	R: AAGCAATTCCGTCTCAACGTGGTC		
*SNAT4*	F: CTGCTTGCTGTGGCAATCCT	119	XM_021092582.1
	R: TTCCCAGGCCATCCAAATGC		
*SLC38A10*	F: CATGTTCGTGATCGTGCTCTCC	168	XM_005668573
	R: TCGTCCAGGCTGTCGTAGGT		
*SOD2*	F: TGCAAGGAACAACAGGTCTGG	188	NM_214127.2
	R: TGATGTACTCGGTGTGAGGC		
*GSH-Px*	F: GCTCGGTGTATGCCTTCTCT	103	NM_214201.1
	R: AGCGACGCTACGTTCTCAAT		
*GCLM*	F: GTGCAGTTGACATGGCCTGC	282	XM_001926378.4
	R: CAGTTAAATCGGGCGGCATC		
*FATP1*	F: GTGCTGGTGATGGACGAACTGG	180	XM_021076151
	R: GCCTGCTTTGCCCTCTACTCCT		
*SLC2A3*	F: TGGGCATCGTCATCGGGATTCT	134	XM_021092392
	R: AAAGGGAAGGGCGGCACAGT		
*SLC2A13*	F: GCTGCGGCGAACAACAAAGGA	167	XM_021092534
	R: AACTGCCCTCCCGTGATGAAGA		
*β-actin*	F: TGGAACGGTGAAGGTGACAGC	177	XM_021086047.1
	R: GCTTTTGGGAAGGCAGGGACT		

### Statistical analysis

All data were initially collated using Excel 2021 and analyzed using the *t*-test in SAS 9.4 for analysis. Normality of the data was assessed with the Shapiro–Wilk statistic (*W* > 0.05). In analyzing the reproductive performance of sows, the sows and their litter size were analyzed as the experimental unit. A Poisson regression analysis was carried out to analyze the number of live born, healthy piglets per litter, weak piglets per litter, mummified and stillborn by using the total born as a covariate and other data were analyzed using the relevant samples of randomly sampled sows from each group as the experimental unit. Mean and SEM were used to express the results of the experiments. When *p* < 0.05, differences were considered significant, while 0.05 ≤ *p* < 0.1 were considered to indicate a tendency.

## Results

### Reproductive performance and feed intake

As shown in Table 3, dietary genistin supplementation significantly increased the average number of live born per litter (*p* < 0.05), and tended to increase the number of healthy piglets per litter (*p* = 0.058), but decreased the average weight of live burns per litter (*p* < 0.05). Dietary genistin supplementation significantly decreased the number of mummified and stillbirths per litter (*p* < 0.05), and significantly increased the total feed intake of sows in gestation and their average daily intake (*p* < 0.05).

**Table 3:** Effects of dietary supplementation with genistin on reproductive performance and feed intake of sows.

Item	Control^1^ (*n* = 31)	Genistin^2^ (*n* = 34)	SEM	*p*-value
Total born, *n*	15.19	16.13	0.73	0.207
Live born, *n*	13.76^b^	15.23^a^	0.63	0.016
Healthy piglets per litter^3^, *n*	12.97	13.83	0.47	0.058
Weak piglets per litter^4^, *n*	0.63	0.70	0.21	0.713
Mummified and stillborn, *n*	1.03^a^	0.52^b^	0.24	0.041
Average litter weight, kg	20.68	21.14	0.76	0.544
Average weight of live born piglets, kg	1.48^a^	1.35^b^	0.05	0.005
Duration of farrowing^5^, min	229.48	202.04	18.11	0.136
Total feed intake, kg	274.00^b^	282.20^a^	1.73	<0.001
ADFI^6^, kg	2.57^b^	2.63^a^	0.02	<0.001

1 Control, sows were fed with a basal diet.

2 Genistin, sows were fed a basal diet supplemented with 150 mg/kg of genistin.

3 Healthy piglets refer to piglets with body weight ≥0.9 kg.

4 Weak piglets refer to piglets with body weight <0.9 kg.

5 The farrowing duration was recorded. The duration of farrowing means the time from the birth of the first piglet to the expulsion of all placentas.

6 ADFI, average daily feed intake.

### Serum hormones and metabolites

As compared to the control group, genistin supplementation tended to decrease the serum concentrations of TC (*p* = 0.081) and LDL-C (*p* = 0.096) at 85 days of gestation (Table 4). Moreover, the serum leptin (LEP) concentration also tended to be decreased in the genistin group (*p* = 0.073).

**Table 4:** Effects of dietary supplementation with genistin on serum hormones and metabolite levels of sows.

Item	Control^1^	Genistin^2^	SEM	*p*-value
Day 35 of gestation				
E2, pmol/L	135.37	140.28	6.41	0.384
PROG, pmol/L	1746.48	1877.01	17.90	0.845
LEP, ng/L	2278.47	2267.55	29.96	0.724
IGF-1, μg/L	19.73	18.84	0.71	0.240
TP, g/L	66.85	68.87	4.71	0.685
ALB, g/L	27.85	28.71	0.85	0.337
GLU, mmol/L	3.03	3.00	0.22	0.915
BUN, mmol/L	3.20	3.34	0.42	0.721
TG, mmol/L	0.33	0.40	0.08	0.408
TC, mmol/L	1.73	1.64	0.24	0.742
HDL-C, mmol/L	0.96	1.00	0.06	0.578
LDL-C, mmol/L	0.89	0.83	0.13	0.659
Day 85 of gestation				
E2, pmol/L	125.70	133.31	4.98	0.158
PROG, pmol/L	1837.96	1796.27	77.78	0.604
LEP, ng/L	2484.88	2414.05	34.90	0.073
IGF-1, μg/L	20.60	21.18	0.38	0.176
TP, g/L	69.23	67.32	3.59	0.663
ALB, g/L	31.74	27.9	2.30	0.126
GLU, mmol/L	3.46	3.32	0.13	0.339
BUN, mmol/L	4.08	3.60	0.38	0.233
TG, mmol/L	0.44	0.47	0.06	0.605
TC, mmol/L	1.63	1.40	0.12	0.081
HDL-C, mmol/L	0.90	0.79	0.11	0.367
LDL-C, mmol/L	0.75	0.65	0.05	0.096

E2, estradiol; PROG, progesterone; LEP, leptin; IGF-1, insulin-like growth factor-1; TP, total protein; ALB, albumin; GLU, glucose; BUN, blood urea nitrogen; TG, triglyceride; TC, total cholesterol; HDL-C, high density lipoprotein-cholesterol; LDL-C, low-density lipoprotein cholesterol.

1 Control, sows were fed with a basal diet.

2 Genistin, sows were fed a basal diet supplemented with 150 mg/kg of genistin.

### Serum immunoglobulins and cytokines

As shown in Table 5, genistin supplementation significantly increased the serum concentrations of IgA, IgG, and IgM at 35 days of gestation (*p* < 0.05). The serum concentrations of IL-10 and IFN-γ were both increased upon genistin supplementation (*p* < 0.05). Moreover, genistin supplementation tended to increase the serum IgM concentration at 85 days of gestation (*p* = 0.098).

**Table 5:** Effects of dietary supplementation with genistin on serum immunoglobulin and cytokines of sows.

Item	Control^1^	Genistin^2^	SEM	*p*-value
Day 35 of gestation				
IgA, μg/mL	40.38^b^	45.27^a^	1.77	0.022
IgG, μg/mL	243.85^b^	289.73^a^	19.28	0.039
IgM, μg/mL	29.54^b^	34.66^a^	1.13	0.002
IL-1, ng/L	75.89	79.69	2.30	0.133
IL-6, ng/L	774.58	838.88	43.75	0.172
IL-10, ng/L	89.88^b^	124.07^a^	8.29	0.002
IFN-γ, pg/mL	1345.37^b^	1886.92^a^	98.97	<0.001
TNF-*α*, pg/mL	273.49	307.36	25.12	0.208
Day 85 of gestation				
IgA, μg/mL	45.40	45.63	1.84	0.904
IgG, μg/mL	258.83	259.84	15.29	0.949
IgM, μg/mL	30.34	35.19	2.43	0.098
IL-1, ng/L	94.45	96.29	5.48	0.744
IL-6, ng/L	890.03	902.54	43.77	0.781
IL-10, ng/L	100.66	110.60	8.29	0.258
IFN-γ, pg/mL	1756.15	1899.31	109.31	0.244
TNF-α, pg/mL	240.89	251.52	7.62	0.206

IgA, immunoglobulin A; IgG, immunoglobulin G; IgM, immunoglobulin M; IL-1, interleukin-1; IL-6, interleukin-6; IL-10, interleukin-10; IFN-γ, interferon-g; TNF-α, tumor necrosis factor-α.

1 Control, sows were fed with a basal diet.

2 Genistin, sows were fed a basal diet supplemented with 150 mg/kg of genistin.

### Antioxidant capacity

As shown in Table 6, dietary genistin supplementation significantly increased the serum concentration of CAT at 35 days of gestation (*p* < 0.05). It has no influences on the serum concentrations of antioxidative enzymes such as the SOD, GSH-Px, and CAT at 85 days of gestation (*p* > 0.05).

**Table 6:** Effects of dietary supplementation with genistin on serum antioxidant indexes of sows.

Item	Control^1^	Genistin^2^	SEM	*p*-value
Day 35 of gestation				
SOD, U/mL	243.69	222.47	24.49	0.407
MDA, nmol/mL	1.78	2.08	0.41	0.482
GSH-Px, U/mL	3655.23	3886.09	251.25	0.389
CAT, U/mL	0.58^b^	1.13^a^	0.20	0.029
T-AOC, U/mL	1.69	0.81	0.61	0.209
Day 85 of gestation				
SOD, U/mL	246.68	239.23	22.64	0.749
MDA, nmol/mL	3.71	3.91	0.69	0.776
GSH-Px, U/mL	4404.07	4612.48	152.00	0.200
CAT, U/mL	1.55	1.17	0.38	0.335
T-AOC, U/mL	2.14	2.25	0.58	0.859

SOD, superoxide dismutase; MDA, malondialdehyde; GSH-Px, glutathione peroxidase; CAT, catalase; T-AOC, total antioxidant capacity.

1 Control, sows were fed with a basal diet.

2 Genistin, sows were fed a basal diet supplemented with 150 mg/kg of genistin.

### Transcriptional abundance of placental genes

As shown in Table 7, genistin supplementation significantly decreased the transcript abundance of *IL-1β* and *H19* in the placental (*p* < 0.05), but increased the transcript abundance of critical imprinting genes such as the *SNTA2*, *SNAT4*, and *IGF-1* (*p* < 0.05). However, transcript abundance of nutrient transporters (e.g., *SLC2A3*, *SLC2A13*, *SLC38A10*, and *FATP1*) did not differ between the two groups (*p* > 0.1).

**Table 7:** Effect of dietary supplementation with genistin on the transcript abundance of placental functional genes *IL1β*, *TNF-α*, *IGF-1*, *IGF-2*, *H19*, *SNAT1*, *SNAT2*, *SNAT4*, *SLC38A10*, *SOD2*, *GSH-Px*, *GCLM*, *FATP1*, *SLC2A13*, and *SLC2A3*.

Item	Control^1^	Genistin^2^	SEM	*p*-value
IL-1β	1.00	0.43	0.13	0.005
TNAF-α	1.00	1.18	0.47	0.706
IGF-1	1.00^b^	2.03^a^	0.15	<0.001
IGF-2	1.00	1.04	0.14	0.755
H19	1.00^a^	0.44^b^	0.14	0.003
SNAT1	1.00	0.91	0.40	0.818
SNAT2	1.00^b^	2.32^a^	0.16	<0.001
SNAT4	1.00^b^	1.5^a^	0.19	0.033
SLC38A10	1.00	1.02	0.39	0.950
SOD2	1.00	0.559	0.23	0.104
GSH-Px	1.00	0.85	0.27	0.604
GCLM	1.00	0.92	0.34	0.809
FATP1	1.00	1.47	0.40	0.262
SLC2A13	1.00	2.01	0.98	0.327
SLC2A3	1.00	1.30	0.33	0.390

IL1-β, interleukin-1 β gene; TNF-α, tumor necrosis factor-α gene; IGF-1, insulin like growth factor 1 gene; IGF-2, insulin like growth factor 2 gene; H19, imprinted gene H19; SNAT1, sodium-coupled neutral amino acid transporter 1, SNAT2, sodium-coupled neutral amino acid transporter 2; SNAT4, sodium-coupled neutral amino acid transporter 4; SLC38A10, solute carrier family 38 member 10; SOD2, superoxide dismutase 2 gene; GSH-Px, glutathione peroxidase; GCLM, glutamate-cysteine ligase modifier subunit gene; FATP1, fatty acid transport protein 1; SLC2A3, solute carrier family 2 member 3; SLC2A13, solute carrier family 2 member 13.

1 Control, sows were fed with a basal diet.

2 Genistin, sows were fed a basal diet supplemented with 150 mg/kg of genistin.

## Discussion

The physiological significance of soy isoflavones has been widely elucidated over the past decade, with genistin, the main component of natural isoflavones, receiving particular attention for its estrogenic, anti-inflammatory and antioxidant properties (24). In the present study, we found that genistin supplementation at a dose of 150 mg/kg during gestation significantly increased the number of live born piglets, which is consistent with previous studies on sows (22). It is noteworthy that genistin supplementation reduced the average weight of live born piglets, which may be attributed to the limited uterine volume. Research has shown that uterine volume during gestation of sows does have an impact on litter size to some extent (25). As a result of the limited uterine volume and increased litter size, piglets may experience reduced birth weight (26). Modern pig breeds are known to have a high ovulation rate, but excessive ovulation in sows can negatively affect embryo development and survival during gestation (27). However, in this study, genistin was found to significantly reduce the number of stillbirths and mummies, corroborating earlier findings in sows (28).

Estrogen and progesterone are two important hormones that regulate reproductive processes in mammals (29). During gestation, it can promote embryo implantation and development, inhibit uterine contraction, and ensure the safe development of the fetus (8, 30, 31). Inadequate estrogen production is more likely to result in fetal loss during gestation (32). Previous research has indicated that dietary daidzein supplementation can result in a significant increase in serum progesterone and estrogen levels by the 35 days of gestation (22). However, this study showed that dietary genistin supplementation had no significant effect on progesterone and estrogen on either 35 days or 85 days of gestation. This discrepancy may be attributed to several factors: (I) distinct mechanisms of action between genistin and daidzein; (II) variances in animal species, dosages, and physiological states. Insulin-like growth factor 1 (IGF-1) has a positive regulatory effect on the supply of fetal glucose, affecting the transport of glucose and amino acids in the placenta, and is also an important factor in determining fetal size (33). IGF-I also plays an important role in controlling skeletal muscle growth (34, 35). In the present study, we found that genistin had no significant effect on serum IGF-1, probably because the 35 days of gestation and the 85 days of gestation were not the peak period of IGF-1 secretion. Studies have shown that the serum concentration of IGF-1 in sows at 10–12 days of gestation is the highest (36). The uterus or placenta of a sow can also produce IGF-1. By detecting the transcript abundance of *IGF-1* gene in placental tissue, it was found that genistin significantly increased the relative expression of *IGF-1*, which was similar to the results of daidzein treatment (22). It is hypothesized that genistin may increase the transfer of maternal nutrients to the fetus and reduce the incidence of mummification and stillbirth by enhancing the expression of the IGF-I gene. Late gestation is an important period of fetal development. During this period, the mother needs to increase feed intake and fat deposition to meet the energy needs of fetal growth. Leptin is a key hormone involved in the body’s regulation of energy balance. A high concentration of leptin acts on the hypothalamus, leading to a decrease in animal feed intake (37). The present study found that genistin had a decreasing trend in serum leptin content on the 85 days of gestation, which may have the effect of promoting feed intake in sows and increasing nutrient supply to the fetus in the later stages of gestation.

Serum metabolites serve as indicators reflecting the nutritional metabolic status in animals. Abnormal lipid metabolism often leads to increased lipid levels and increases the susceptibility of polyunsaturated fatty acids to free radical oxidative damage, thereby affecting the reproductive performance of animals (38). Studies have shown that TC and LDL-C are related to the occurrence of preterm birth, TC concentration is negatively correlated with the occurrence of preterm birth, and high concentration of LDL-C in the third trimester of pregnancy may also lead to the occurrence of preterm birth (39, 40). In this experiment, by measuring the concentration of serum metabolites, it was found that dietary genistin supplementation trend to decrease the total cholesterol (TC) and low-density lipoprotein cholesterol (LDL-C) in serum metabolites of pregnant sows on the 85 days. It is concluded that genistin may reduce the incidence of miscarriage by reducing serum TC and LDL-C concentrations.

Oxidative stress in animals is often accompanied by inflammatory response, which is characterized by over-expression of pro-inflammatory factors and activation of the immune system (41). In the rat model of myocardial ischemia-reperfusion injury, it was found that 40–60 mg/kg genistin could significantly reduce the levels of TNF-α, IL-6 and IL-8 in serum (42). Another experiment showed that different doses of genistin significantly reduced the production of TNF-α, IL-1β and IL-6 in serum of rats (43). IL-10 is an anti-inflammatory factor and IFN-γ is a lymph factor with a wide range of immunomodulatory effects. In the present study, we found that genistin could significantly increase the levels of IL-10 and IFN-γ in the serum of sows on the 35 days of gestation. At the same time, it was found that genistin could significantly increase the content of IgA, IgM and IgG in the serum of sows during the 35 days of gestation, and IgM in the serum of sows on the 85 days of gestation had an increasing trend. These are similar to the results that dietary supplementation of 40 mg/kg genistein can significantly increase the content of IgM and IgG in the serum of broilers at 21 days of age (44), and diets were supplemented with 200 mg/kg daidzein can significantly increase the content of IgG in the serum of sows on the 35 days of gestation (22). Therefore, it is speculated that genistin can reduce inflammation and improve immunity by increasing the content of IL-10, IFN-γ, IgA, IgM and IgG in serum, so as to improve the reproductive performance of sows.

In the gestation stage, due to the rapid development needs of the fetus and envir that genistin has a scavenging effect on superoxide anion (O_2_^−^) and 200 μM concentration is equivalent to 0.08 U/mg superoxide dismutase (45). In the rat model of myocardial ischemia-reperfusion injury, it was found that 40–60 mg/kg genistin onmental factors, it may cause metabolic disorders in pregnant sows and produce excessive free radicals in the body, resulting in oxidative stress in rat (46, 47). Free radicals can be scavenged by antioxidant enzymes such as SOD, GSH-Px and CAT (48). Genistin has been proved to have antioxidant properties. Previous studies have shown genistin could significantly increase the concentration of CAT, SOD and GSH and significantly reduce the concentration of MDA in serum (42). In the present study, we found that genistin supplementation could significantly increase the concentration of CAT in the serum of sows on the 35 days of gestation, indicating that genistin could also play an antioxidant role in sows. Therefore, genistin may improve the antioxidant capacity in early pregnancy and improve the reproductive performance of sows.

The placenta has a crucial role in fetal growth by facilitating the exchange of gases, nutrients, and metabolites between the mother and the fetus (49). Both pro-inflammatory and anti-inflammatory cytokines are produced by the placenta, and the overproduction of these cytokines, such as IL-1β, is linked to the development of gestational complications (50). Studies have found that IL-1β is more capable of inducing apoptosis of fetal membrane cells than IL-6, and then affecting fetal development (51). The present study found that dietary genistin supplementation significantly decreased the transcript abundance of *IL-1β* in the placenta of sows. It is inferred that genistin can reduce the fetal inflammatory response by reducing the transcript abundance of *IL-1β* gene in the placenta, thereby reducing the fetal death. *H19* and *IGF-2* are maternal and paternal imprinted genes, respectively. Placental imprinted genes can regulate placental nutrient transport and are the main regulators of placental development and fetal growth. In general, maternally imprinted genes inhibit fetal growth, while paternal imprinted genes promote fetal growth (52–54). In the present study, we found that genistin supplementation significantly reduced the transcript abundance of *H19* gene in placental tissue of sows, but had no significant effect on the transcript abundance of *IGF-2* gene, indicating that genistin may affect the transcript abundance of placental imprinting gene *H19* gene to reduce the inhibition of fetal growth. Both *SNAT2* and *SNAT4* are amino acid transporter genes that are crucial for transporting both essential and non-essential amino acids from the mother to the fetus (55). In the current study, genistin supplementation significantly increased the gene expression of *SNAT2* and *SNAT4* in placental tissue. Therefore, it is presumed that genistin can aid in the deposition and growth of fetal proteins by facilitating the transfer of amino acids from the mother to the fetus. In summary, genistin may promote fetal growth and ensure fetal health by affecting the transcript abundance of inflammatory genes, placental imprinting genes and nutrient transport genes to improve the reproductive performance of sows.

## Conclusion

In conclusion, these results suggest that genistin can be used as an effective additive to improve the reproductive performance of sows. The benefits of genistin supplementation may be attributed to enhanced immune and antioxidant capacity, as well as regulation of placental essential genes.

## Data Availability

The original contributions presented in the study are included in the article/supplementary material, further inquiries can be directed to the corresponding authors.
